# Validation of In-House Imaging System via Code Verification on Petunia Images Collected at Increasing Fertilizer Rates and pHs

**DOI:** 10.3390/s24175809

**Published:** 2024-09-06

**Authors:** Kahlin Wacker, Changhyeon Kim, Marc W. van Iersel, Mark Haidekker, Lynne Seymour, Rhuanito Soranz Ferrarezi

**Affiliations:** 1 Department of Horticulture, University of Georgia, Athens, GA 30602, USA; kahlin.wacker@uga.edu (K.W.); mvanier@uga.edu (M.W.v.I.); 2 Department of Plant Science and Landscape Architecture, University of Connecticut, Storrs, CT 06269, USA; changhyeon.kim@uconn.edu; 3 College of Engineering, University of Georgia, Athens, GA 30602, USA; mhaidekk@uga.edu; 4 Department of Statistics, University of Georgia, Athens, GA 30602, USA; seymour@uga.edu

**Keywords:** plant image segmentation, chlorophyll fluorescence, normalized difference vegetation index

## Abstract

In a production environment, delayed stress recognition can impact yield. Imaging can rapidly and effectively quantify stress symptoms using indexes such as normalized difference vegetation index (NDVI). Commercial systems are effective but cannot be easily customized for specific applications, particularly post-processing. We developed a low-cost customizable imaging system and validated the code to analyze images. Our objective was to verify the image analysis code and custom system could successfully quantify the changes in plant canopy reflectance. ‘Supercascade Red’, ‘Wave© Purple’, and ‘Carpet Blue’ Petunias (*Petunia* × *hybridia*) were transplanted individually and subjected to increasing fertilizer treatments and increasing substrate pH in a greenhouse. Treatments for the first trial were the addition of a controlled release fertilizer at six different rates (0, 0.5, 1, 2, 4, and 8 g/pot), and for the second trial, fertilizer solution with four pHs (4, 5.5, 7, and 8.5), with eight replications with one plant each. Plants were imaged twice a week using a commercial imaging system for fertilizer and thrice a week with the custom system for pH. The collected images were analyzed using an in-house program that calculated the indices for each pixel of the plant area. All cultivars showed a significant effect of fertilizer on the projected canopy size and dry weight of the above-substrate biomass and the fertilizer rate treatments (*p* < 0.01). Plant tissue nitrogen concentration as a function of the applied fertilizer rate showed a significant positive response for all three cultivars (*p* < 0.001). We verified that the image analysis code successfully quantified the changes in plant canopy reflectance as induced by increasing fertilizer application rate. There was no relationship between the pH and NDVI values for the cultivars tested (*p* > 0.05). Manganese and phosphorus had no significance with chlorophyll fluorescence for ‘Carpet Blue’ and ‘Wave© Purple’ (*p* > 0.05), though ‘Supercascade Red’ was found to have significance (*p* < 0.01). pH did not affect plant canopy size. Chlorophyll fluorescence pixel intensity against the projected canopy size had no significance except in ‘Wave© Purple’ (*p* = 0.005). NDVI as a function of the projected canopy size had no statistical significance. We verified the ability of the imaging system with integrated analysis to quantify nutrient deficiency-induced variability in plant canopies by increasing pH levels.

## 1. Introduction

Imaging is a widely used approach to measure plant health due to its rapidity, non-invasive nature, and capacity to reveal plant or crop spatial fluctuations. There are assorted commercial systems with optimized cameras that serve well for many applications, which often provide good images but have limited customization. In research, custom analysis is often desired to fulfill specific research parameters. For analyzed indices, normalized difference vegetation index (NDVI) and chlorophyll fluorescence intensity are selected as imaging paradigms to monitor plant health.

NDVI is an index used in several different fields of research for plants. It originated with Rouse et al. [1] as a way of using spectral reflectance to quantify plant area. Due to the strong difference in NDVI output, this gained much traction and is still used for quantifying plant area, density, and health in remote sensing [2]. In this application, it is used more as a Boolean green/not green comparison averaged over an area. In our application, we removed the non-plant areas from our imaging and quantified the distribution over the plant area alone, which gave us more nuanced insight into the plant status than can be derived without background removal. NDVI is one of many indices used for plant health, though it is the most common [2]. Chlorophyll fluorescence imaging captures fluorescence emitted by chlorophyll in the plant. When supplied with actinic light, chlorophyll fluoresces at wavelengths greater than 650 nm [3]. That allows a camera equipped with a long-pass filter (>650 nm) and a shorter wavelength light spectrum (usually blue light) for excitation to produce a chlorophyll fluorescence image. The imaging consists of exposing the plant to blue light while a long-pass filter filters out all but the red/far-red light produced by chlorophyll fluorescence, which is then recorded by the image sensor [4]. This is representative of the photosynthetically active parts of the plant and can show damage symptoms before visible effects appear [5].

As used in field applications with remote sensing, controlled environment agriculture can and has benefitted from imaging as a quantitative regulatory mechanism [6]. In these fields, the “remote” part of the imaging tends to be much closer, where an imaging system can be mounted to an irrigation boom or a stationary element of the facility that the plants move past.

Fertilizer impacts canopy nutrient status and these variations are the core factors imaging systems identify. Depending on the plant’s nutritional and health status, the optical properties will change. Nutrient deficiencies have different effects, and quantifying those to identify a need for change is sought after in the industry for control and optimization [2].

pH is a critical component of plant nutrient availability for both macro- and micronutrient availability [7,8]. Because of this, substrate pH is a heavily monitored and controlled part of both conventional and hydroponic horticultural production. pH measurement and regulation are challenging and are a high-stakes problem in commercial growth circles. In light of these challenges, imaging provides a fast, non-destructive way to evaluate plant health, as the plant is the most critical part of the system, and analyzing it for weaknesses allows for remedial actions to occur if needed in the growing system. pH is unstable in hydroponic systems in waters with low carbonate and bicarbonate levels. The effects of the roots and unanticipated chemical interactions cause variations that often require frequent checking and remediation, as very small changes can have large effects [8,9].

Detecting physiological responses to changes in pH and fertilizer application often requires destructive tissue analyses, which can be expensive and time-consuming. A non-destructive approach, such as image-based phenotyping, could be a cost-effective way to detect these symptoms in an objective and high throughput manner. There are a few options for doing this using imaging. Commercial systems using RGB and multispectral cameras can be procured, often with some limitations. The hardware capabilities may be satisfactory, but the analysis might need to be customized for specific research objectives, such as creating indices using particular wavelengths. Photochemical reflectance index (PRI) is another common index used in multispectral plant analysis that typically uses images at 531 and 570 nm [10], and neither the implemented commercial system nor the system developed has those specific wavelengths, so a researcher seeking PRI would have to find a new imaging system, order it custom, or add it for themselves on a post-processing step performed separately. Adhikari and Nemali [11] and Stamford et al. [12] started using commercial systems and realized it was not adequate for their research goals and ended up developing their own independent systems. We followed the same trend and produced our customized image analysis program to fit our research needs because the commercial system we used had a hardware weakness where the focus correction of the infrared image was warped and shifted, thus requiring correction in the analysis code before NDVI could be used. In a research environment, the details of a process can be very important for understanding and providing trustworthy results, and the internal software was not forthcoming about the processes involved in the analysis; in addition, we desired specific parameters and graphical outputs that the internal software was not able to provide. Also, image-based phenotyping systems can also use hyperspectral cameras with machine learning algorithms, which are not easily accessible to plant scientists due to their complexity and high cost.

The objective was to verify that the image analysis code could successfully quantify the changes in plant canopy reflectance from images collected by a commercial imaging system and to induce variability in plant canopies by irrigating at several pH levels to cause micronutrient deficiencies to verify the ability of the imaging system to quantify physiological responses. We hypothesize that imaging has the potential to identify times of concern needing remediation to reduce the frequency of real effects on plant health. This trial was conceived to induce canopy variation by micro- and macronutrient deficiencies to verify the total functionality of the imaging system and quantify pH-based canopy changes in petunias.

## 2. Materials and Methods

This research was structured to have two studies processed in parallel, with the first study (fertilizer trial) being imaged with a commercial system and analyzed using our algorithm, and the second study (pH trial) being imaged and analyzed using our in-house algorithm.

### 2.1. Fertilizer Trial

#### 2.1.1. Location and Experimental Conditions

The plants were grown and maintained in a greenhouse at the University of Georgia (College of Agricultural and Environmental Sciences, Department of Horticulture, Horticultural Physiology Laboratory) in Athens, GA, from 21 January until 26 March 2023, at the termination of the experiment. The greenhouse presented an average daily light integral of 17.8 ± 7.4 mol m^−2^ d^−1^, temperature of 22.5 ± 0.8 °C, and vapor pressure deficit of 1.5 ± 0.4 kPa (mean ± standard deviation).

#### 2.1.2. Plants

Petunias (*Solanaceae Petunia* × hybridia) of three different cultivars, ‘Supercascade Red’, ‘Wave© Purple’, and ‘Carpet Blue’ (Ball Premier, Chicago, IL, USA), were chosen due to known susceptibility to nutrient changes and planted in 10 cm pots (500 mL volume). The growing media used was a soilless substrate (Metro-Mix^®^ 830; SunGro Horticulture, Agawam, MA, USA). Seeds were sown on 8 December and germinated in a vertical farm at 25 °C and 800 mg/L CO_2_ and 34.6 mol m^−2^ d^−1^ photosynthetic photon flux density until 21 January 2022, when they were transplanted into individual pots and placed in the greenhouse.

#### 2.1.3. Treatments

Fertilizer treatments were selected across a wide range of accepted rates to induce variability in the treatments, and were the addition of a 19N-1.8P-6.6K controlled release fertilizer (19-4-8, Harrells, Lakeland, FL, USA) at six different rates, specifically, 0, 0.5, 1, 2, 4, and 8 g per pot, or g per 500 mL of media. Eight replicates of each treatment per cultivar were planted and imaged.

#### 2.1.4. Imaging Acquisition

From 1 through 26 March 2022, plants were imaged twice weekly using a commercial imaging system (Topview; Aris B.V., Eindhoven, The Netherlands).

#### 2.1.5. Experimental Design and Statistical Analysis

The experimental design included six treatments of increasing fertilizer rate application and eight plants per treatment per cultivar, each representing a replication (*n* = 8). Statistical analysis was performed using a polynomial regression with R^2^ (quality of line fit) and *p*-value (probability of no effect of the independent variable on the dependent variable) for the regression fit in statistical software (SigmaPlot version 11.0; Systat Software, San Jose, CA, USA). The lines shown are to visualize trends and are not quantitative models.

### 2.2. pH Trial

#### 2.2.1. Location and Experimental Conditions

The plants were grown and maintained in a greenhouse at the University of Georgia (College of Agricultural and Environmental Sciences, Department of Horticulture, Controlled Environment Agriculture lab) in Athens, GA, from 20 September until 6 October 2023, at the termination of the experiment. These plants were grown in peat plugs put into 7.62 × 7.62 × 6.35 cm rockwool blocks with a hole (Delta 4; Grodan, Roermond, The Netherlands) to serve as a substrate for hydroponic fertigation. Treatments at pH levels of 4.0, 5.5, 7.0, and 8.5 were maintained in the fertigation reservoirs. The greenhouse conditions were, on average, a daily light integral of 18.5 ± 3.6 mol m^−2^ d^−1^, a temperature of 23.4 ± 0.4 °C, and a vapor pressure deficit of 0.9 ± 0.1 kPa (mean ± standard deviation).

#### 2.2.2. Plants

Petunias of three different cultivars, ‘Supercascade Red’, ‘Wave© Purple’, and ‘Carpet Blue’ (Ball Premier, Chicago, IL, USA) chosen due to known susceptibility to nutrient changes and planted in a plug tray, using a soilless substrate (Metro-Mix^®^ 830; SunGro Horticulture, Agawam, MA, USA). Seeds were sown on August 31 and germinated in a vertical farm at 25 °C and 800 mg/L CO_2_ and 34.6 mol m^−2^ d^−1^ photosynthetic photon flux density until September 20, when they were transplanted into 7.62 × 7.62 × 6.35 cm rockwool cubes with holes (Delta 4; Grodan, Roermond, The Netherlands) and placed in the greenhouse, where they were sub-irrigated three times per day with a pH modified mix of 15N-2.2P-12.4K water-soluble fertilizer (Jack’s Professional^®^ LX 15-5-15 Cal-Mag LX; JR Peters, Allentown, PA, USA). Two previous trials were attempted with a soilless substrate (Metro-Mix^®^ 830; SunGro Horticulture, Agawam, MA, USA) though the desired substrate pH levels were unreachable by the calcium carbonate applications available, and thus the transition to fertigation in an inert substrate.

#### 2.2.3. Treatments

pH treatments were selected across a wide range of accepted rates to induce variability in the treatments, and included adding phosphoric acid and potassium hydroxide to reach pH levels in each of the four fertigation reservoirs of 4.0, 5.5, 7.0, and 8.5, from a starting pH of an average of 6.35. These pH levels were amended each time images were collected. From 20 September through 6 October 2023, plants were imaged thrice a week using the imaging system developed by the lab.

The end data we used were the NDVI, chlorophyll fluorescence intensity, average and projected canopy size from the imaging system, and the same tissue analysis as from the fertilizer trial.

#### 2.2.4. Image Acquisition

The images were obtained with our in-house system from 20 September through 6 October 2023. This system illuminates light-emitting diodes (LEDs) of single wavelengths, takes a picture, saves that image, and then loops through the other wavelengths of the LEDs in the system. We only use red, infrared, blue, green, and chlorophyll fluorescence images.

#### 2.2.5. Experimental Design and Statistical Analysis

There were four treatments of increasing pH and eight plants per treatment per cultivar, each representing a replication (*n* = 8). The statistical analysis was a polynomial regression yielding a *p*-value and R^2^ fit to the regression in statistical software (SigmaPlot 11.0; Systat Software, San Jose, CA, USA). The lines are to visualize trends and are not quantitative models. This methodology was chosen as a visual aid as these studies were not designed to be concrete data on stimulus/response reactions of petunia.

### 2.3. Imaging Analysis for Both Trials

**Figure 1:**
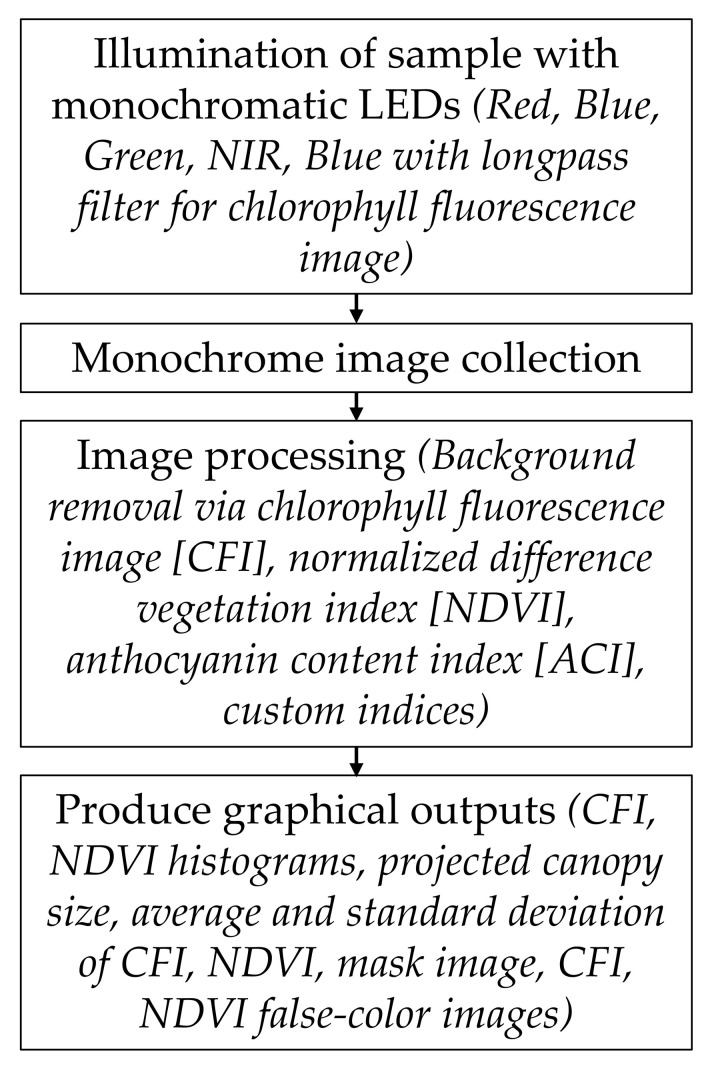
Flowchart diagram of the in-house imaging system to capture and analyze plant images under different light-emitting diodes (LEDs) wavelengths using chlorophyll fluorescence imaging to calculate spatial NDVI and canopy size per pixel for detailed plant analysis.

The collected images were analyzed using an in-house program that calculated the NDVI for each pixel of plant area. To maximize the chance of showing significant treatment differences, a mask was obtained showing just those pixels with functioning chlorophyll. This was performed using chlorophyll fluorescence imaging, which is the process whereby blue light is applied to the imaging area, and an image is taken through a long-pass filter, which only transmits light at wavelengths longer than 665 nm. Chlorophyll fluoresces, making the photosynthetically active material visible and identifying the pixels in the NDVI image to be used for the analysis. This allows the plant area to be isolated and spatial NDVI to be calculated on a per-pixel basis. A false-color image can be generated showing the NDVI values. The average and standard deviation of the plant NDVI can be extracted, as well as from other indexes and each wavelength image, including the chlorophyll fluorescence image. Projected canopy size can be extracted from the images as well, using the filtering and mask image process where the number of pixels of the plant area is summed, and this represents the 2-dimensional structure of the plant as viewed from above (Figure 1).

**Figure 2:**
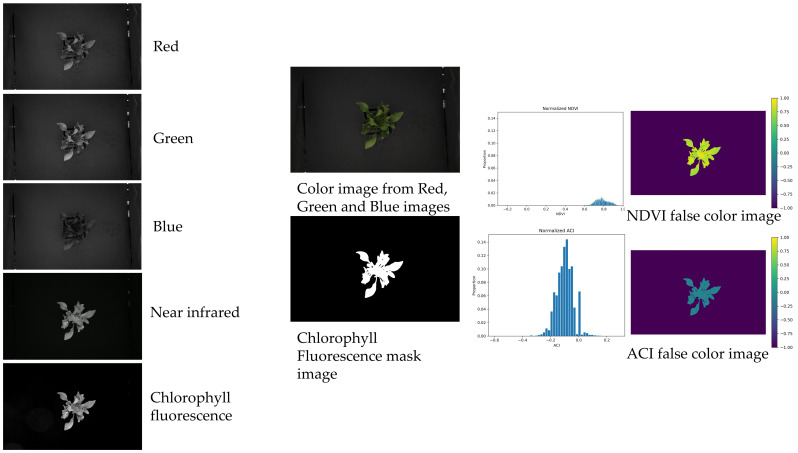
Details of each image obtained by the imaging system, histogram representation, and normalized difference vegetation (NDVI) and anthocyanin content index (ACI) false color images.

The graphical outputs resulted in multiple parameters exemplified in Figure 2 and listed in the measurements section.

### 2.4. Measurements

The measurements taken and correlated were NDVI, chlorophyll fluorescence, and projected canopy size from the images, and after harvest, the dry weight after oven-drying, and plant tissue mineral concentration analysis by Waters Agricultural Laboratories (Camilla, GA, USA). Nitrogen, phosphorus, potassium, magnesium, and calcium were analyzed, with the nitrogen being quantified by combustion at high temperature as outlined in Nelson and Sommers [13], and the remaining concentrations are evaluated by wet acid digestion in nitric acid and hydrogen peroxide as defined by Twyman [14], then quantified by inductively coupled plasma atomic emission spectrometer (ICP-AES).

## 3. Results

### 3.1. Fertilizer Trial

**Figure 3:**
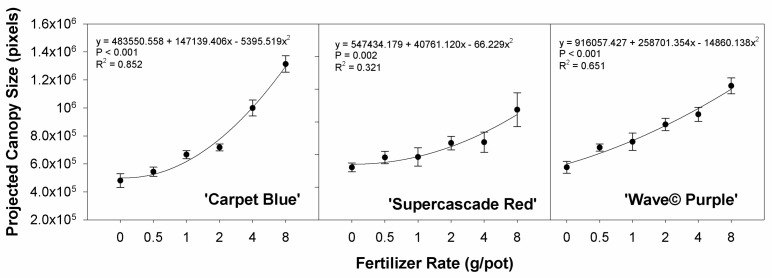
Projected canopy size of three cultivars of petunia (*Solanaceae Petunia* × hybridia) grown under increasing fertilizer rates. The fertilizer rate applied has a significant effect on the two-dimensional area of the plant, as measured by a commercial imaging system and analyzed by our in-house software. Each point is the mean of 8 replicates with standard error bars.

The projected canopy size is the two-dimensional projection of the three-dimensional canopy of the plant that gives a quick, non-destructive analog of biomass. All cultivars show a significant effect of fertilizer on projected canopy size, with ‘Carpet Blue’, ‘Supercascade Red’, and ‘Wave© Purple’ (*p* < 0.01, Figure 3). The relationships have a positive trend, which is anticipated with the higher rates of available nutrients, the growth rates were higher.

**Figure 4:**
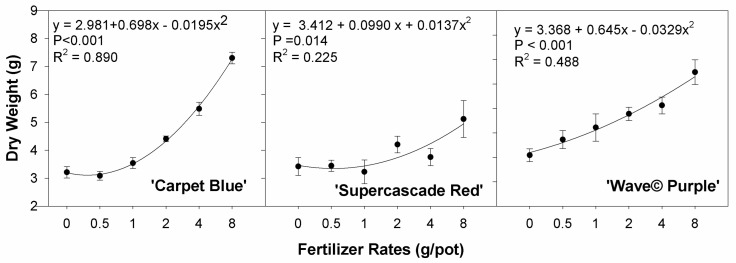
Dry mass of three cultivars of petunia (*Solanaceae Petunia* × hybridia) grown under increasing fertilizer rates. All cultivars show significance in the treatments. Each point is the mean of 8 replicates with standard error bars.

All three cultivars showed significance between the dry weight of the above-substrate biomass and the fertilizer rate treatments for ‘Carpet Blue’, ‘Supercascade Red’, and ‘Wave© Purple’ (*p* < 0.01, Figure 4). The curvature is positive, as anticipated with increased nutrient availability there is increased biomass.

**Figure 5:**
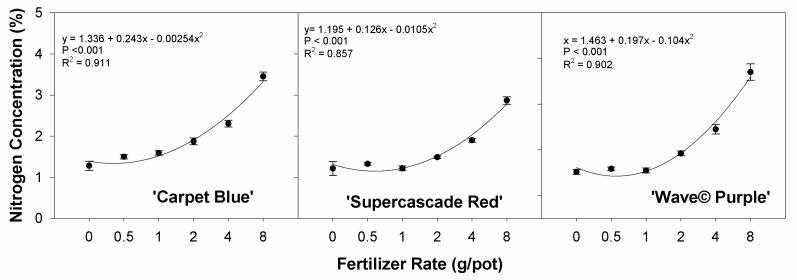
Nitrogen concentration as a function of increasing fertilizer rate on three cultivars of petunia (*Solanaceae Petunia* × hybridia). The nitrogen concentration was shown to be significantly related to the fertilizer rate. Each point is the mean of 8 replicates with standard error bars.

Plant tissue nitrogen concentration as a function of the applied fertilizer rate has a significant positive curvature for all three cultivars (*p* < 0.001, Figure 5). This represents the variable nitrogen uptake rates in the plants based on the applied fertilizer, thus showing the efficacy of the treatments in terms of the actual effects of the treatments.

**Figure 6:**
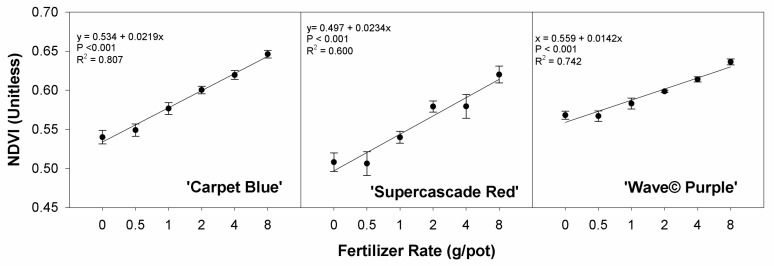
Normalized difference vegetation index (NDVI) from the imaging system for three cultivars of petunia (*Solanaceae Petunia* × hybridia) at increasing fertilizer application rates. The normalized difference vegetation index (NDVI) responses are shown to be significantly related to fertilizer application. Each point is the mean of 8 replicates with standard error bars.

The graphed quadratic fit between the plant average of NDVI and the treatment of fertilizer rate in Figure 6 shows the direct relationship between the two and the ability to use spatial NDVI to evaluate and identify suboptimal conditions, with these being defined by an NDVI lower than the best-case scenario (around 0.65 for ‘Carpet Blue’, 0.63 for ‘Supercascade Red’, and 0.64 for ‘Wave© Purple’) (*p* < 0.001, Figure 6).

**Figure 7:**
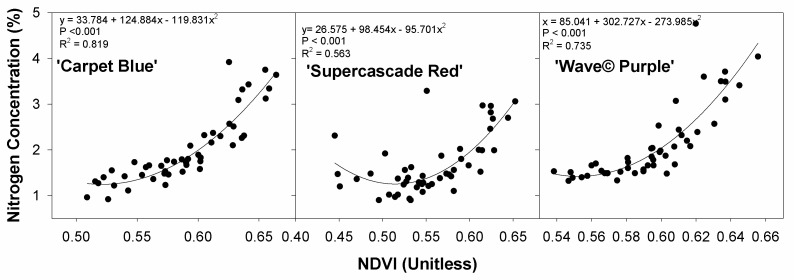
Plant tissue nitrogen concentration as a function of the average pixel normalized difference vegetation index (NDVI) of the plant area for three cultivars of petunia (*Solanaceae Petunia* × hybridia) subjected to increasing fertilizer rates.

The relationship between the NDVI average pixel intensity and the plant tissue nitrogen concentration shows that nitrogen concentration is significantly related to the fertilizer rate (*p* < 0.001, Figure 7). The NDVI is also significantly associated with the nitrogen concentration for all cultivars. This is a positive curvature as anticipated, with nitrogen increasing quadratically with average NDVI, and with nitrogen being associated with increased plant health like NDVI.

**Figure 8:**
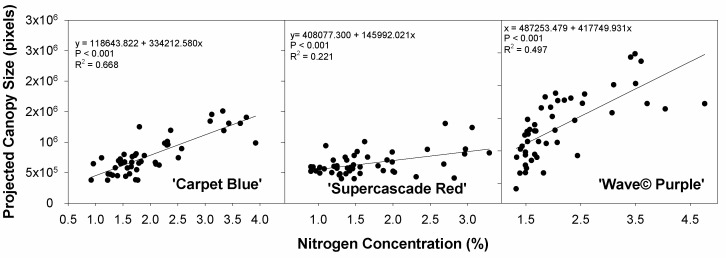
Projected canopy size in pixels against the tissue nitrogen concentration for three cultivars of petunia (*Solanaceae Petunia* × hybridia) grown at increasing fertilizer rates. Primarily, this shows the effect of nitrogen concentration on the plant growth size.

The effect of nitrogen concentration on the projected canopy size follows a primarily positive curvature except for ‘Wave© Purple’, though all three cultivars show significance in the relationship (*p* < 0.001, Figure 8). This shows the effect of the nitrogen concentration on the growth of the plants, with the nitrogen being affected by the fertilizer treatments.

**Figure 9:**
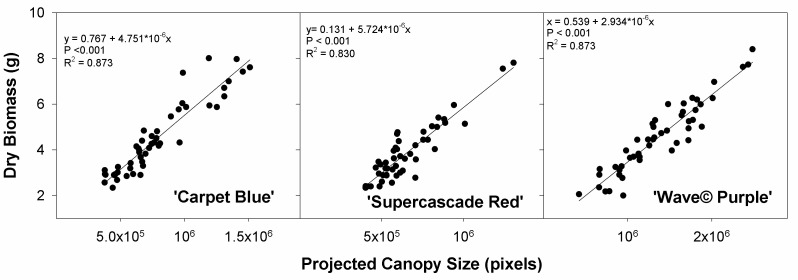
Dry biomass as a function of the projected canopy size for three cultivars of petunia (*Solanaceae Petunia* × hybridia) grown at increasing fertilizer rates. This shows the correlation between the imaged plant size and the dry mass.

The correlation between the plant dry biomass and the projected canopy size as recorded by the imaging system shows a significant correlation for all three cultivars (*p* < 0.001, Figure 9). This shows the capability of an imaging system to show plant size and, thereby, plant growth. It also shows that the two-dimensional projection of the canopy size, at least in these three petunia cultivars, is a good representation of plant size.

**Figure 10:**
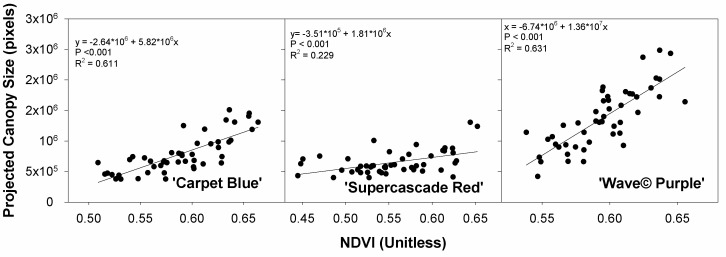
Projected canopy as a function of normalized difference vegetation index (NDVI), both from imaging system for three cultivars of petunia (*Solanaceae Petunia* × hybridia) grown at increasing fertilizer rates.

The canopy size plotted against the NDVI has a positive curvature, which represents increased plant health, with the NDVI and plant size being the desired maxima (*p* < 0.001, Figure 10). These quadratic relationships were all significant, and the trends were positive as anticipated, with higher values of each representing increased plant health.

### 3.2. pH Trial

**Figure 11:**
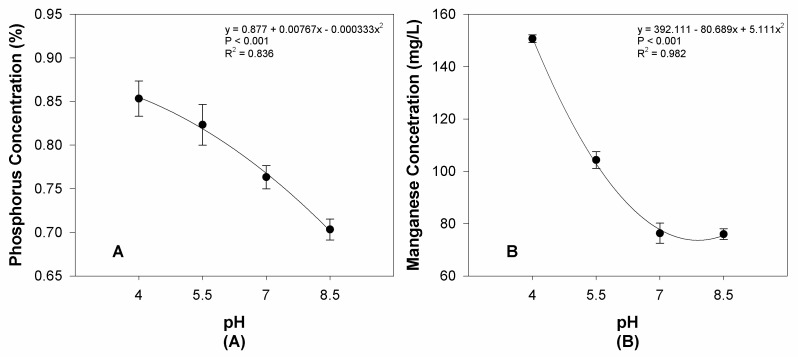
(**A**) Phosphorus and (**B**) Manganese concentrations of ‘Supercascade Red’ in response to increasing pH. These nutrient decreases in the plant tissue were the desired effect in the experiment to display deficiencies or other visible symptoms. Each point is the mean of 8 replicates with standard error bars.

The availability responses of phosphorus and manganese vary in increasing pH-amended solutions (*p* < 0.001, Figure 11A and Figure 11B, respectively). The values in the graph show the concentration and content of ‘Supercascade Red’ (Figure 11). Differing pH values change the bioavailability of various substances, and this is a graphical representation of the uptake of these two nutrients in this study.

**Figure 12:**
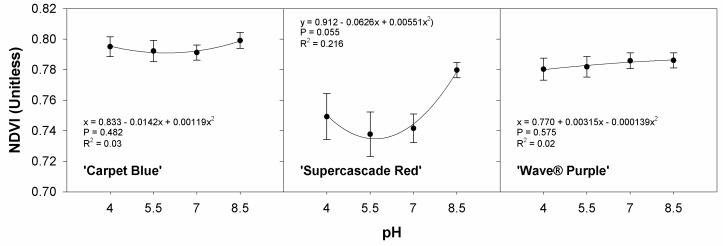
Normalized difference vegetation index (NDVI) response to pH for three cultivars of petunia (*Solanaceae Petunia* × hybridia) grown in increasing pH solutions. Normalized difference vegetation index (NDVI) did not show a meaningful response to pH, except for ‘Supercascade Red’, which could be considered significant due to several extreme outliers. Each point is the mean of 8 replicates with standard error bars.

The response of spatial NDVI in the plant canopies should, in theory, reflect the pH treatments, as the pH treatments alter the bioavailability of nutrients in the solution. We found no significant relationship between the pH and the NDVI values likely due to conflicting effects at the different pH levels with the different availability dynamics in play (*p* > 0.05, Figure 12). ‘Supercascade Red’ could be considered significant due to a few considerable outliers, the potential correction in future research would render the results significant. This could explain the unanticipated relationship shown in ‘Supercascade Red’ with its parabolic response to pH.

**Figure 13:**
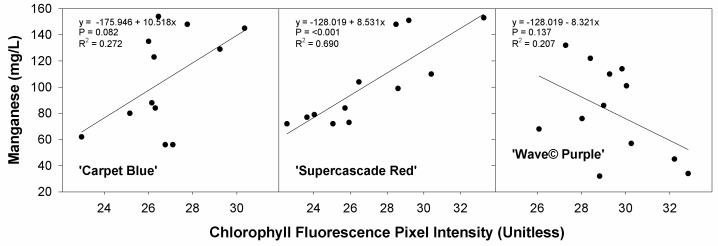
Manganese content against chlorophyll fluorescence for three cultivars of petunia (*Solanaceae Petunia* × hybridia) grown in increasing pH solutions. There was no significant effect of Manganese on image-measured parameters on ‘Carpet Blue’ and ‘Wave© Purple’ cultivars.

The relationship between the average chlorophyll fluorescence and the manganese content shows the effect of the manganese content on canopy reflectance. There is no significant correlation between the manganese and chlorophyll fluorescence for ‘Carpet Blue’ and ‘Wave© Purple’ (*p* > 0.05, Figure 13), though ‘Supercascade Red’ was found to have significance (*p* = 0.011). This represents an improvement in the correlations found against NDVI.

**Figure 14:**
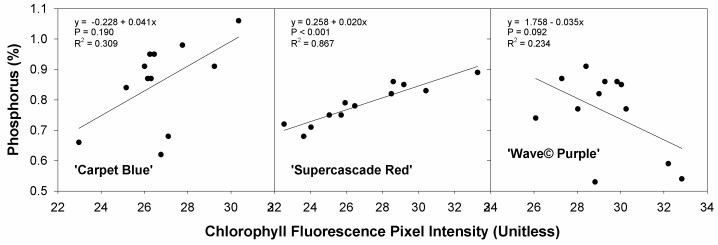
Phosphorus concentration against chlorophyll fluorescence for three cultivars of petunia (*Solanaceae Petunia* × hybridia) grown in increasing pH solutions. Stronger effect with phosphorus, explained by phosphorus being a macronutrient rather than a micronutrient.

The relationship of a nutrient against chlorophyll fluorescence, specifically the phosphorus concentration, is very similar to Figure 12. The correlations are much stronger, with ‘Carpet Blue’ and ‘Wave© Purple’ still being insignificant (*p* > 0.05) and ‘Supercascade Red’ being significant (*p* < 0.001, Figure 14). This is due to phosphorus being a macronutrient.

**Figure 15:**
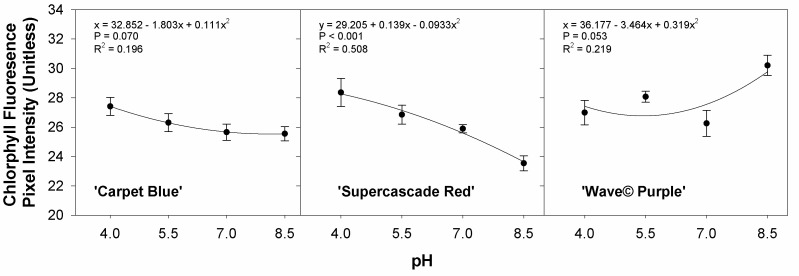
Average chlorophyll fluorescence pixel intensity as a function of pH for three cultivars of petunia (*Solanaceae Petunia* × hybridia) grown in increasing pH solutions. Each point is the mean of 8 replicates with standard error bars.

Figure 15 shows all the effects of the various nutrients as represented by the chlorophyll fluorescence intensity against pH treatments. Significance is found in ‘Supercascade Red’ only (*p* < 0.001, Figure 15). This shows the effect of micro- and macronutrient availability, and the stronger relationship to pH, with the ability to quantify this effect using imaging.

**Figure 16:**
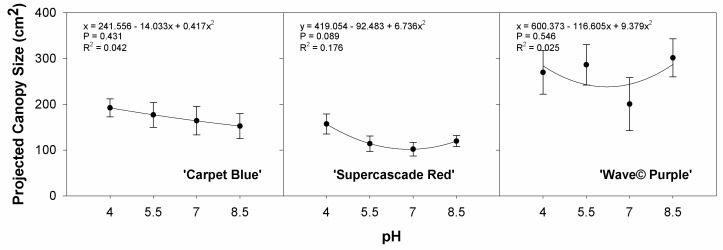
Projected canopy size as a function of the pH treatments for three cultivars of petunia (*Solanaceae Petunia* × hybridia) grown in increasing pH solutions. Each point is the mean of 8 replicates with standard error bars.

Figure 16 shows the projected canopy size graphed against the pH treatments. Plant size is one of the factors in plant health status that is optimized for, thus knowing plant size as a function of the pH serves as a powerful indicator of plant health, though in this case, none of the cultivars showed statistical significance (*p* > 0.05, Figure 16).

**Figure 17:**
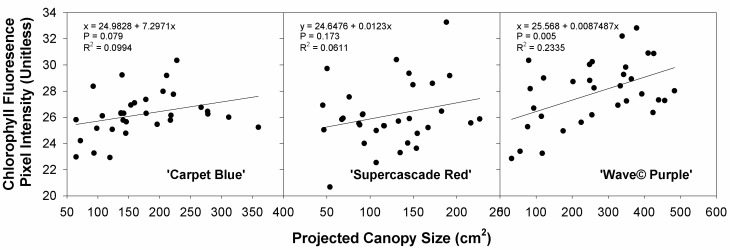
Chlorophyll fluorescence as a function of the projected canopy size for three cultivars of petunia (*Solanaceae Petunia* × hybridia) grown in increasing pH solutions.

The average chlorophyll fluorescence pixel intensity against the projected canopy size represents the relationship between size and the chlorophyll fluorescence intensity, with which there is not a significant quadratic fit except in ‘Wave© Purple’ (*p* = 0.005, Figure 17). These all have poor R^2^ values, too, limiting the ability to predict them from an equation. This graph was meant to show some of the ability to interpret plant health in multiple parameters. Plant size is a commonly prioritized factor in plant vigor, and this was attempted to see if chlorophyll fluorescence intensity could also be used, though only ‘Wave© Purple’ had any significance.

**Figure 18:**
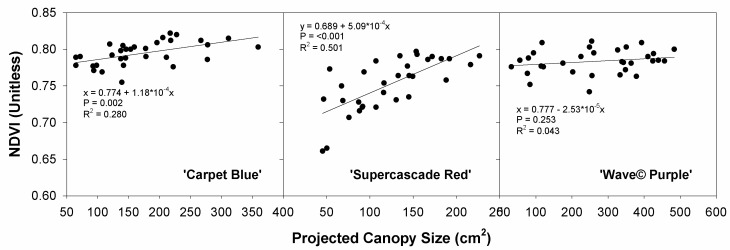
Normalized difference vegetation index (NDVI) plotted against the projected canopy size for three cultivars of petunia (*Solanaceae Petunia* × hybridia) grown in increasing pH solutions.

Figure 18 shows NDVI plotted as a function of the projected canopy size. This falls under the same criteria of plant health being often paired with size. The purpose was to compare plant size with chlorophyll fluorescence to gauge the ability to predict these together, but the values were not significant (*p* > 0.05, Figure 18).

## 4. Discussion

### 4.1. Fertilizer Trial

This trial was conceived to apply variable nutrient stress to petunias by increasing fertilizer application rates. The projected canopy size was positively correlated with the applied fertilizer rates. Plants require nutrients, which can be limiting, leading to reduced plant growth. When nutrients are in suitable supply, plants grow optimally, and the size will increase at the greatest rate. With reduced fertilizers, plant sizes and growth rates are decreased. This is consistent for potted petunias within the fertilizer range applied for the experiment [15] and accepted practice and theory of plant nutrition [16].

We found that the final biomass increased with the fertilizer treatments, indicating that the fertilizer rates applied affected plant growth. As the plant grows, and due to the multidimensional nature and the constituent complex shapes inherent therein, it can be difficult to quantify whole plant growth precisely. Most accurate quantifications of true plant canopy size are made using a leaf area meter, which must be fed leaf by leaf through the device. This is time-consuming and destructive. Rather than quantifying the whole leaf area, we measured the projected canopy size using the imaging system. It correlated well with the total final biomass and allowed an analog of true plant size to be collected quickly and nondestructively. As these are both measurements of plant size and were shown to be affected by the fertilizer rate, as explained above, this correlates well with the theory of both fertilizers positively influencing plant size as well as the two- and three-dimensional growths being correlated.

Size is only an outward effect of inward processes, and from the data collected from plant tissue analysis, the nitrogen concentration increased significantly with the applied fertilizer rate, indicating one of the potential causes of the increased plant size per fertilizer rate. As nitrogen is one of the essential macronutrients for plant growth [16], increased nitrogen concentration, being significantly related to increased plant size, is anticipated by accepted plant nutrition theory. This is also consistent with the findings of Cabrera-Bosquet et al. [17], who looked at the NDVI of wheat in pots in a greenhouse and drew its relationship with the nitrogen content and area of the wheat plants and found a similar strong connection between NDVI and nitrogen content and NDVI and plant area. NDVI is one of the parameters the imaging system can extract and one of the metrics whose value is in question, the NDVI was graphed against the fertilizer rate, which we have seen is tied to the plant size and nitrogen concentration. These were strongly related again, allowing us to consider using NDVI as an indicator of plant stresses. NDVI, representing the pigmentation of the plant and plants commonly showing stress symptoms in foliar color change, should be a good indicator of plant responses [16]. NDVI was then plotted against the nitrogen concentration, also with significant results, allowing for potential estimation of that parameter. Nitrogen is one of the essential macronutrients [16] and thus plays a large role in plant health and, thereby, canopy pigmentation. This is a desired and tested capability in multiple fields showing good promise, with Edalat et al. [18] finding similar effects in corn to the findings of Cabrera-Bosquet et al. [17]. Edalat et al. [18] used corn in the field and found NDVI connected to the nitrogen concentration.

The projected canopy size was plotted against nitrogen concentration, the final dry mass, and the NDVI, with significance in all situations. The significance of projected canopy size with nitrogen is consistent with essential macronutrients affecting plant growth [16]. The data indicate that as the fertilizer rates increase, the NDVI as measured by the imaging system increases significantly, showing the imaging system’s potential to give insight into the plant’s status.

‘Supercascade Red’ showed less dependence on nitrogen concentration, as an effect of a more reduced response to fertilizer treatments than the other two cultivars. This is interesting, as in the following trial, ‘Supercascade Red’ is far more sensitive than ‘Carpet Blue’ and ‘Wave© Purple’.

Similarly to the relationship found in the fertilizer rates and the projected canopy size and the applied fertilizer rate and the nitrogen in the plant, the projected canopy size and the nitrogen show significance (*p*-values of <0.001, 0.011, and <0.001, respectively, for ‘Carpet Blue’, ‘Supercascade Red’, and ‘Wave© Purple’). This response is well-known since nutrients play an important role in metabolic processes and are key components of the photosynthetic pathways and result in adequate, vigorous growth when occurring without nutrient deficiencies. This is consistent with Alem et al. [15], who found that with the increased fertilizer, and thus increased nitrogen, there was increased plant growth, which is consistent with the theory behind nitrogen being an essential macronutrient [16], with that theory being that more nitrogen means increased plant growth.

Biomass and projected canopy size were positively related to fertilizer treatments. The curvature for all three cultivars is indicative of plant structure, as the biomass is the sum of the dry matter in the plant and the projected canopy size is the two-dimensional projection of the plant area, making the relationship a reflection of the three-dimensional structure of the plant with vertical components representing an increase in biomass without an increase in projected canopy size. With both being measurements of the plant size, this relationship is consistent with other studies reported in the literature [19].

The relationships between NDVI and projected canopy size were not exclusively positive, though Cabrera-Bosquet et al. [17] found similarly that the NDVI and the projected canopy size were related, if not in a direct linear fashion. The driving factor behind this phenomenon is the condition of plants being richer in nutrients, resulting in larger plant size and fewer deficiency symptoms, which are indicated by higher NDVI values. The concept was to make the computationally easier projected canopy size measurement and use that to interpret plant health. The relationships are not strong enough to use this indicator to predict NDVI, but if paired with a plant growth model, it could be useful for interpreting when a plant is growing below optimum, whereas the NDVI can identify canopy effects of nutrient stresses.

### 4.2. pH Trial

This trial was a proof of concept for our in-house imaging system, using pH to control nutrient availability and otherwise stress the plants. To verify if the treatments had an effect, the tissue nutrient content was correlated with the pH treatments. This resulted in differences in phosphorus and manganese, which were chosen to verify the treatment effects. These were found by Gillespie et al. [5] to decrease with increasing pH in plant tissue in basil. Other than the crop difference, both trials used rockwool as a substrate in a greenhouse setting. To evaluate the effectiveness of NDVI in reflecting plant status, this was graphed against the pH treatments, with no significance found. This is likely due to the lack of effect of pH on petunia [20]. This shows the differences between cultivars, where we find near significance in ‘Supercascade Red’ but not in the other two cultivars. The imaging system was capable of quantifying NDVI, but treatments did not impact the NDVI results. In light of the common effects of pH on nutrient availability, in most cases, there should be NDVI differences induced by the pH, though not in petunia. NDVI is used in other crops to map alkaline soil distributions, in one case, in soybeans [21]. In particular, the two nutrients tracked, manganese and phosphorus, both have visible deficiency effects that should be highlighted by NDVI measurement, specifically with phosphorus deficiency showing in leaves turning purple and manganese with leaves experiencing chlorosis [16].

As pH is a driver for limitations in nutrient availability [22] and NDVI is a measurement of plant response to nutrient availability, in an optimal theoretical situation, NDVI as a function of pH should have a strong relationship. This trial did not find significance in the relationship between the pH treatment and the NDVI, which may have been due to the pH-resistant nature of petunia [20] or due to the limited size of the plants, the nutrient requirements were still met by the pH-adjusted fertigation solution.

Chlorophyll fluorescence intensity, as one of the indices of the imaging system, was also evaluated against the treatments. This related more strongly to the treatments (Figure 15). Phosphorus and manganese were then regressed with the chlorophyll fluorescence intensity and showed a better relationship than against NDVI (data not shown). This may be because NDVI measures the pigmentation and coloring of canopy material, whereas chlorophyll fluorescence intensity quantifies the fluorescence of chlorophyll in the leaves. Manganese content has been shown to influence the chlorophyll concentration in tomatoes, with both high and low levels of manganese shown to have a negative effect [23]. Chlorophyll fluorescence intensity was then graphed against the pH treatments, showing significance in ‘Supercascade Red’ but not in the other two cultivars. This suggests multiple interplaying effects, as the NDVI had no relationship, and the chlorophyll fluorescence intensity had a limited relationship [24] with pH. This may be partly due to NDVI being tied to coloration and chlorophyll fluorescence intensity as a representation of chlorophyll activity.

As size is an often prioritized parameter [25], projected canopy size was graphed against the pH treatments, the NDVI, and chlorophyll fluorescence intensity, with the only significant relationship being in ‘Wave© Purple’ for size and chlorophyll fluorescence intensity. We thus see that size is not a good indicator for the pH effects as seen in Smith et al. [20] or with the chlorophyll fluorescence intensity, which is a limited indicator of plant health and nutrient availability, because petunias demonstrated resistance to the different pHs tested during this experiment.

Chlorophyll fluorescence intensity was not significantly correlated with manganese content in this trial except for ‘Supercascade Red’. Manganese has been shown to have both a positive and negative effect on chlorophyll content [23], depending on the rate. In this situation, with the limited response of the petunia to the pH effects, the manganese had a small impact on the chlorophyll, which was only shown with significance in the ‘Supercascade Red’, which is not altogether unexpected.

Chlorophyll fluorescence intensity as a function of the phosphorus concentration was the same as the manganese, with significance found only in ‘Supercascade Red’. Phosphorus, being an essential macronutrient [16], was anticipated to have a stronger effect, though the pH tolerance of petunia mitigated this [20]. Phosphorus normally has such an effect on plants that it can be quantified by chlorophyll fluorescence means [26], though that was not found in this trial except for ‘Supercascade Red’.

Chlorophyll fluorescence intensity versus pH had significance only in ‘Supercascade Red’, which has been a common theme in this process. Given that multiple nutrients are limited in a trial of this type, there may be various effects that may be expressed as constructive or destructive interference. In this instance, with the pH resistance of petunia [20], the probable answer is the reduced effect of the pH treatments. There should be multiple visual effects, such as color changes and chlorosis in nutrient-deficiency situations [16], which did not manifest.

With canopy size being desirable for scalability [22], situations that decrease the size without other benefits are opportunities for improvement and mitigation. To check for an anticipated decrease in plant growth by pH treatment, the relationship between pH and canopy size, which is correlated with the plant biomass, was logged. This relationship showed no significance, likely another artifact of the pH resistance of petunia [20].

Chlorophyll fluorescence intensity as a function of the canopy size had no significance except for in ‘Wave© Purple’, the lack of significance is again the impunity with which petunias adapt to pH treatments [20]. Theoretically, these should correlate as increasing chlorophyll should lead to increased plant size, though this effect was not visible in this study.

NDVI correlated with canopy size had no significance. A plant with a high NDVI should be a plant experiencing minimal stresses and as such should be maximally sized, though we did not see this effect, as cited above, due to the reduced pH effect on petunia [20].

## 5. Conclusions

We verified that the image analysis code successfully quantified the changes in plant canopy reflectance as induced by increasing the fertilizer application rate as well as the ability of the imaging system with integrated analysis to be able to quantify nutrient deficiency-induced variability in plant canopies by increasing pH levels.

## Data Availability

All data are available upon reasonable request.
